# Postmortem chest computed tomography for the diagnosis of drowning: a feasibility study

**DOI:** 10.1080/20961790.2018.1557386

**Published:** 2019-02-23

**Authors:** Junqi Jian, Lei Wan, Yu Shao, Donghua Zou, Ping Huang, Zhuoqun Wang, Ningguo Liu, Yijiu Chen

**Affiliations:** a Shanghai Key Laboratory of Forensic Medicine, Shanghai Forensic Service Platform, Academy of Forensic Science, Shanghai, China; b Department of Forensic Medicine, Soochow University, Suzhou, China

**Keywords:** Forensic sciences; forensic pathology; postmortem CT, drowning, lung 3D reconstruction, CT data

## Abstract

It may be difficult to distinguish the cause of death in drowning cases without specific findings. The aim of this study was to explore the forensic value of thoracic postmortem computed tomography (PMCT) using routine images and three-dimensional (3D) image reconstructions. The imaging data of PMCT examinations of six drowning cadavers, aged 21–54 years, were analyzed. Twelve victims of sudden death from coronary artery disease (CAD) were chosen as a control group. After 3D bilateral lung images were reconstructed using image processing software, an interactive medical image control system was used to measure and analyze parameters including lung volume, lung volume ratio, mean CT value of the whole lung, and lung CT value distribution curves. Lung volume and lung volume ratio were used to assess the shape changes of the lung. Lung CT value distribution curves showed the corresponding number of pixels of the different CT values in the lung image. Lung volume was not significantly larger in drowning cases (mean 2 958 cm^3^) than in controls (mean 2 342 cm^3^). Lung volume ratio values in the drowning group (mean 0.3156) were greater than those in the control group (mean 0.2763); (*P* = 0.02). There was no significant difference between the drowning and control group in the mean CT value of the whole lung. There were differences between lung CT value distribution curves in drowning victims and controls, with drowning victims showing a single peak and CAD cases showing a bimodal distribution. Thoracic PMCT is helpful for the forensic medical diagnosis of drowning. Lung volume ratio and lung CT value distribution are potential indicators to distinguish between drowning and CAD.

## Introduction

In 2014, the World Health Organization released a global report on drowning [1]. Drowning is the third leading cause of unintentional injury death, accounting for 7% of all injury-related deaths. An estimated 372 000 people died from drowning, making drowning a major public health problem worldwide. The autopsy diagnosis of drowning is still difficult [2]. When a victim is suspected of drowning, though important to determine cause of death, the postmortem changes are non-specific [2,3].

In current forensic practice, the diagnosis of drowning is based on autopsy findings, histological findings and the diatom test [2]. These findings are not exclusive to drowning, so other causes of death (disease, injury, poisoning, etc.) still need to be excluded. When the cadaver has undergone putrefaction or tissue dissolution, the difficulty of forensic investigation is increased. Internationally, some new methods have been proposed, such as electrolyte concentration tests [4,5], other microbial tests in water [6,7], and immunohistochemical analysis [8,9]. However, these methods are still difficult to be popularized in practice.

In recent years, postmortem computed tomography (PMCT) has been considered a useful tool in diagnosing the cause of death [10,11]. It may provide a new methodology to diagnose drowning. In previous studies on PMCT imaging in drowning, some findings had high sensitivity but low specificity [12,13]. One is pulmonary changes (ground glass opacity, GGO). Usui et al. [14] evaluated the features of drowning postmortem chest CTs and classified these features into six types: two main types were GGOs with thickened pulmonary interstitium and a centrilobular distribution of ill-defined nodules along the airways. However, they did not compare this finding with other causes of death as controls.

In the present study, postmortem pulmonary CT data including lung volume, lung volume ratio, mean CT value of the whole lung, and lung CT value distribution curves were generated and analyzed from three-dimensional (3D) data. Compared with another cause of death, coronary artery disease (CAD), the authors aimed to define lung changes specific to drowning.

## Materials and methods

### Cases

The authors retrospectively reviewed 15 cases of drowning that had undergone PMCT scanning at the Academy of Forensic Science in China between June 2015 and March 2018. In nine of these 15 cases, the cadavers had sustained serious postmortem decomposition when they were retrieved from the water. The remaining six cadavers retrieved from fresh water (three males, three females), ranging in age from 21 to 54 years, were enrolled in this study. A conventional autopsy had been performed by two board-certified forensic pathologists. The macroscopical signs of typical drowning were found such as lung emphysema, oedema aquosum, Paltauf’s spots, and froth in the trachea. Besides, other causes of death, such as disease, injury, poisoning, were also excluded. The cause of death determined as typical drowning by the autopsy findings and circumstantial evidence from the police investigation. The control group consisted of 12 cases, all of whom experienced sudden death from CAD. The postmortem delay was varying from 5 to 12 h in drowning and the control.

### PMCT

The time interval between PMCT scanning and the conventional autopsy was approximately 2 h. The body was scanned using a 40-detector multisection CT system (Definition AS; Siemens Healthineers, Erlangen, Germany). Raw data were acquired using the following settings: voltage 120 kV, current 240 mAs, collimation 6.0 mm × 1.0 mm. Image reconstruction was achieved at section thicknesses of 5.0 and 0.625 mm, each with an increment of half the slice thickness (soft tissue and bone-weighted reconstruction kernels).

### Lung 3D reconstruction

**Figure 1.**
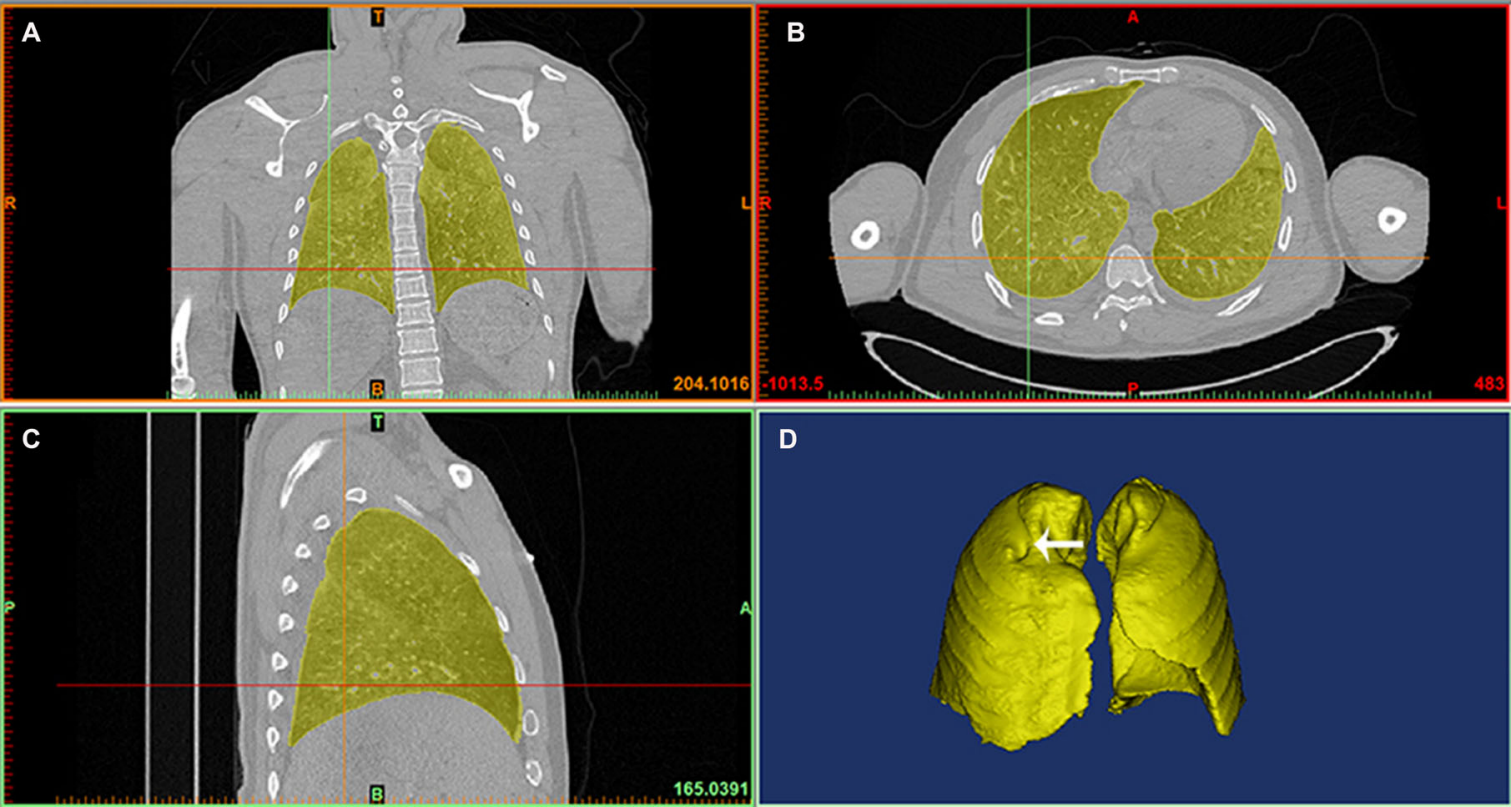
Screen images of 3D reconstructions of lungs with image analysis software. Views in coronal (A), transverse (B) and sagittal (C) planes show the lungs (yellow). The indentation by the rib (white arrow) can be seen on the surface of the 3D lung model (D) due to hyperinflation in drowning.

CT scan data in DICOM format were imported into a commercial software programme (Mimics 19.0 software^®^, Materialise Inc., Leuven, Belgium). 3D bilateral lung image reconstructions were performed, and all images were reviewed using this software. The lungs were segmented semi-automatically with Hounsfield unit (HU) thresholds from −1 000 to 0 HU (Figure 1). In order to measure accurately, the tracheal and main bronchial areas were removed from the images. The segmented image was called the “mask”, a volume of interest including an entire lung. Based on the contiguous sections of CT imaging, lung 3D model was calculated from the mask. The image reconstruction was performed by a single operator.

### Parameter measurements from lung PMCT reconstruction

#### Lung volume

**Figure 2.**
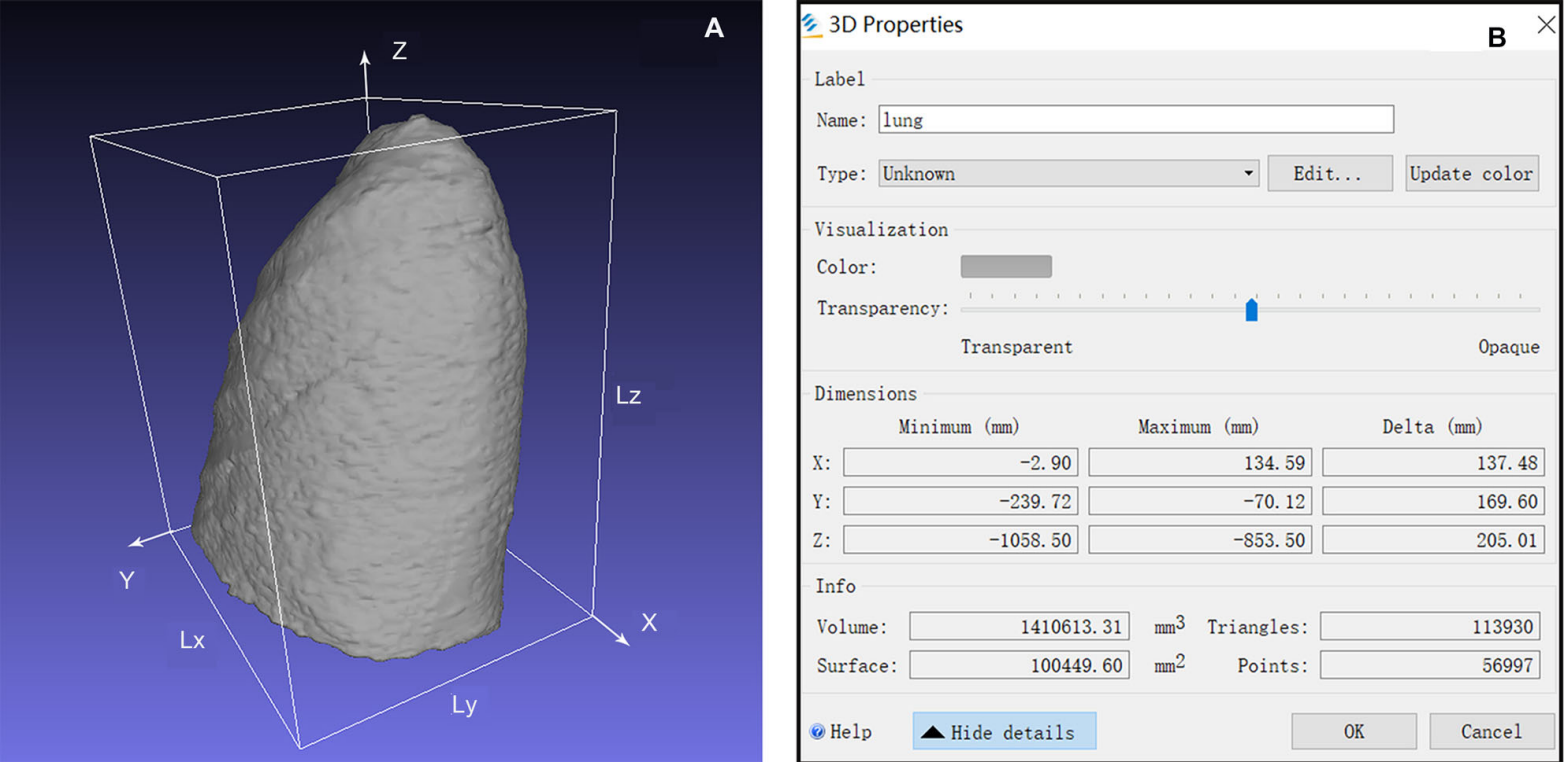
The maximum length of the single lung. (A) A 3D reconstruction of a lung. (B) This software estimated the maximum dimensions of the lung in the transverse, sagittal, and coronal planes, 137.48, 169.60 and 205.01 mm, respectively.

After the 3D image was generated, the software estimated lung volume automatically and stored the results. The maximum dimensions of a single lung were estimated in the transverse, sagittal and coronal planes, named Lx, Ly and Lz, respectively (Figure 2).

#### Lung volume ratio

We attempted to use lung volume ratio (lung volume/maximum volume) to evaluate the degree of inflation of the lung indirectly. The maximum volume is expressed as the product of the maximum dimensions in the transverse, sagittal and coronal planes:

(1) Maximum volume=Lx×Ly×Lz.

#### Lung CT value and distribution curves

When the mask was created, the mean CT value (in Hounsfield units = HU) of the pixels in the whole lung was calculated and stored to create a lung CT dataset, which was imported into another software programme (OriginPro^**®**^, Originlab Corp., Northampton, MA, USA), where the pixel value distribution of the whole lung was expressed in graphical format. In the graph, the X-axis depicts the HU value, and the Y-axis depicts the corresponding number of pixels. Mean distribution curves for both drowning and control groups were created. This function estimated the average number of pixels of different HU values in the individual cause of death. The mean distribution curves reflected the influence of cause of death on lung CT value and weakened the effect of individual factors to some extent.

The radiological data analyses were performed by two forensic pathologists and two radiological specialists. Student’s *t*-test by SPSS Version 19.0 Statistical Package (IBM, Armonk, NY, USA) was used to examine the significance of the differences between the groups. *P* < 0.05 was considered significant.

## Results

### Lung volume

Lung volume was estimated automatically by the CT image analysis software from the 3D generated images. Mean lung volume was larger in the drowning (2 958 cm^3^) than in the control group (2 342 cm^3^), but not to a significant degree (Table 1).

**Table 1.** Lung volume and CT value of the whole lungs in drowning and the control group.

Items	Drowning group (*n* = 6)		Control group (*n* = 12)		*P*-value
	Mean	SD	Mean	SD	
Lung volume (cm^3^)	2 958	882	2 342	443	NS
CT value (HU)	−530	79	−490	100	NS

SD: standard deviation; NS: not significant ; CT: computed tomography.

### Lung volume ratio

**Figure 3.**
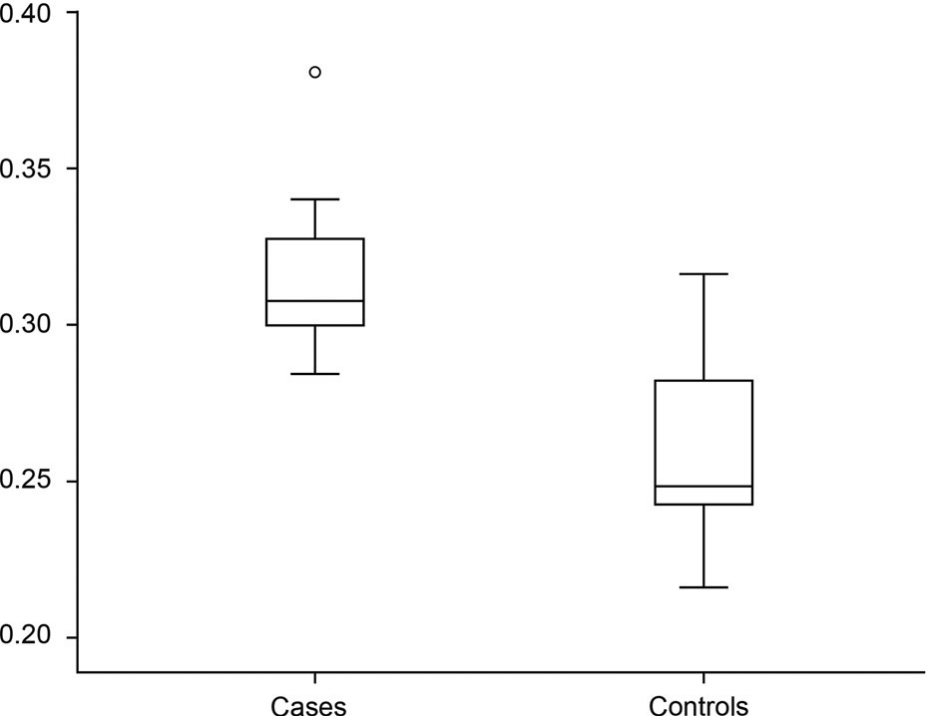
Lung volume ratio. The mean ratio was 0.3156 (±0.0264, *n* = 12) in drowning and 0.2763 (±0.0353, *n* = 24) in coronary artery disease. There was a significant difference between the two groups (*P* = 0.02).

“Lung volume” and “the maximum volume” were estimated based on the unilateral lung. The mean lung volume ratio (Figure 3) 0.3156 (±0.0264, *n* = 12) in the drowning group was significantly greater than that 0.2763 (±0.0353, *n* = 24) found in the control group (*P* = 0.02).

### Lung CT value and distribution curves

The mean lung CT value in the drowning group was not significantly different from that in the control group (Table 1).

**Figure 4.**
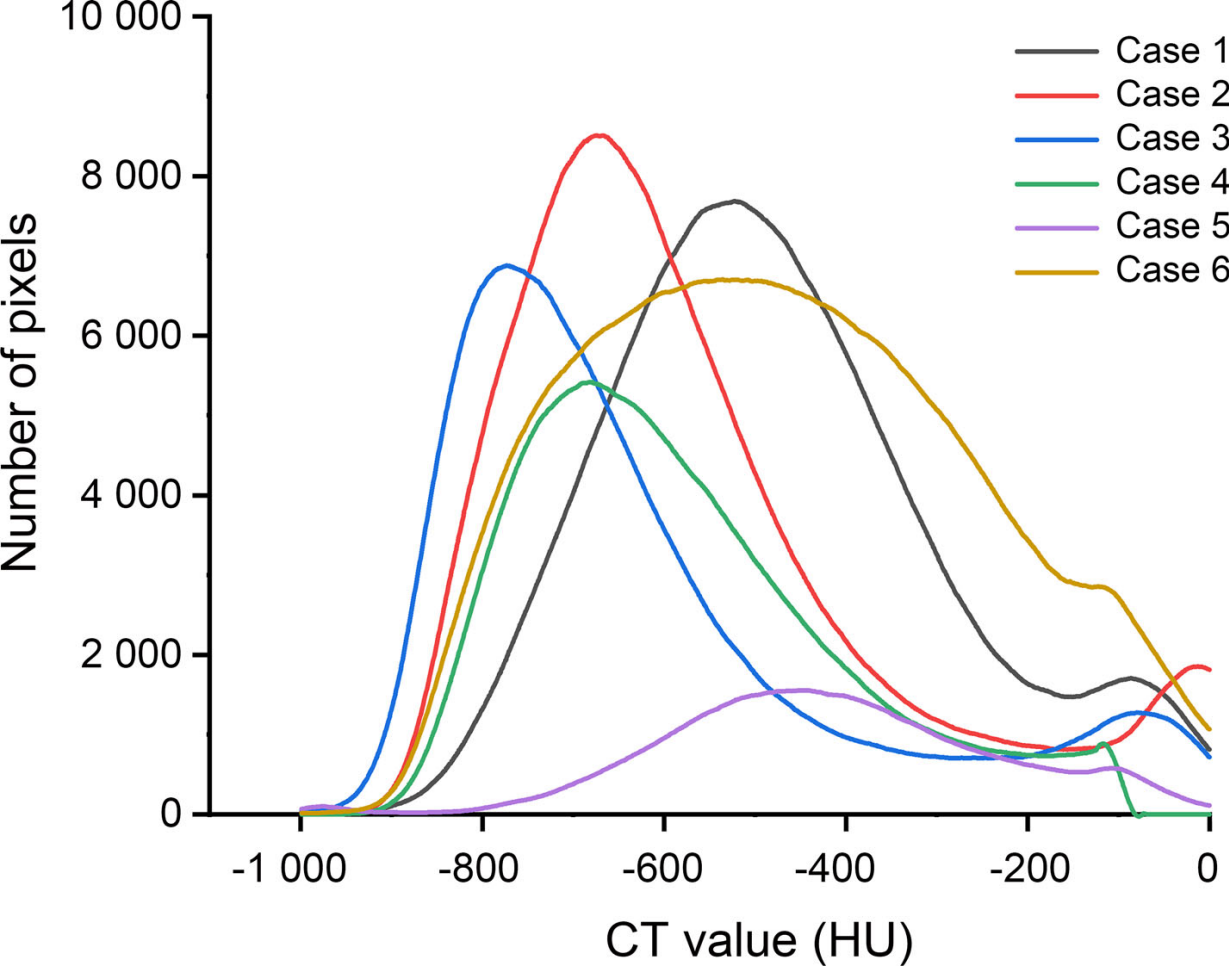
Lung CT value distribution curves in drowning. Most cases presented a similar bell-shaped curve.

**Figure 5.**
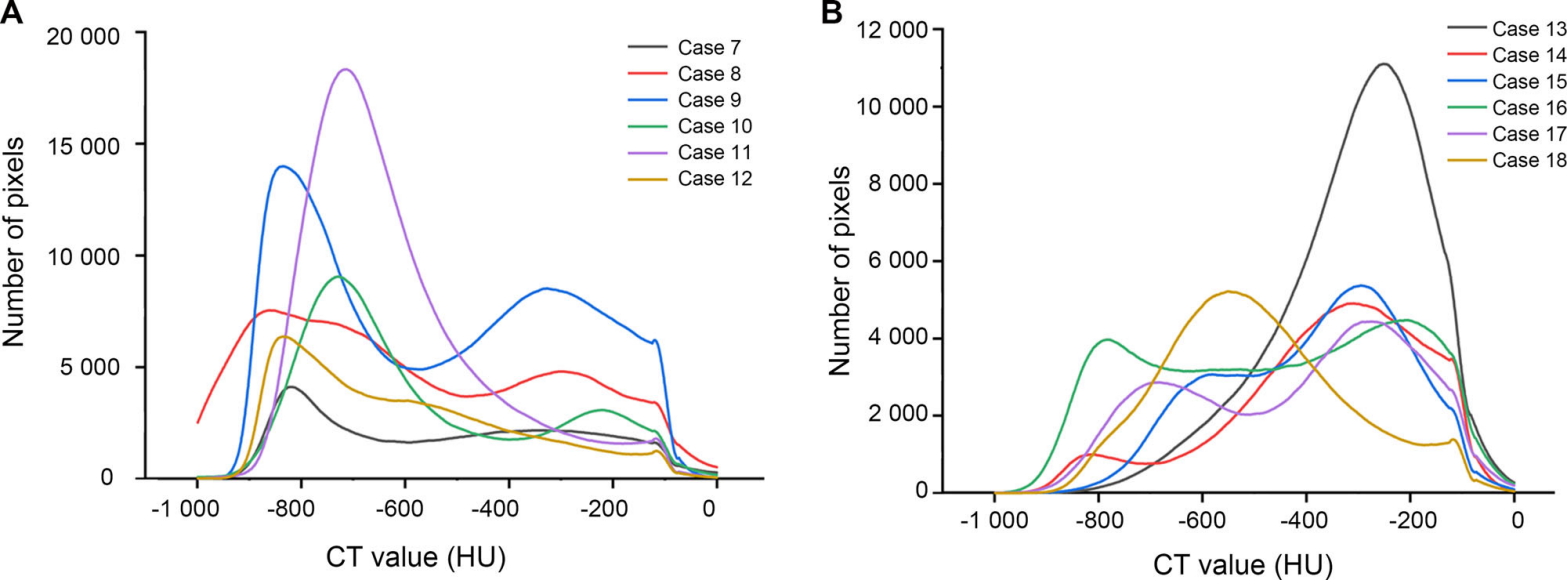
Lung CT value distribution curves in coronary artery disease. (A) The mean CT value of the whole lung is <−500 HU. (B) The mean CT value of the whole lung is >−500 HU. Most of the curves showed two lower peaks between −900 and −700 HU and between −300 and −150 HU. For Cases 7 and 12, the curves showed a long flat and a slow decrease, respectively. The curves presented a similar bell-shaped curve in Cases 11, 13 and 18.

**Figure 6.**
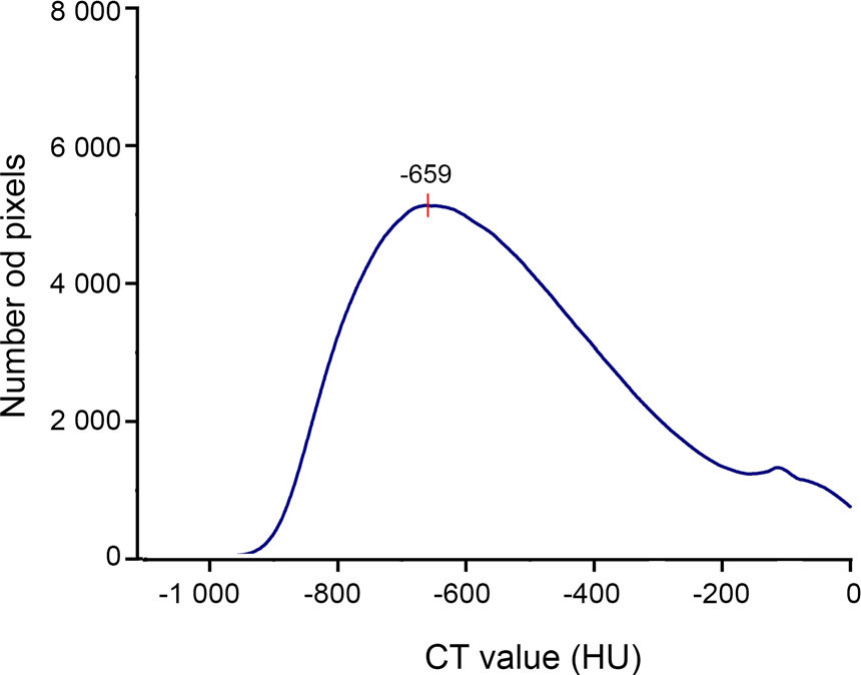
The mean distribution curves in drowning. There was a peak at −659 HU. The number of pixels at the peak was 5 123.

**Figure 7.**
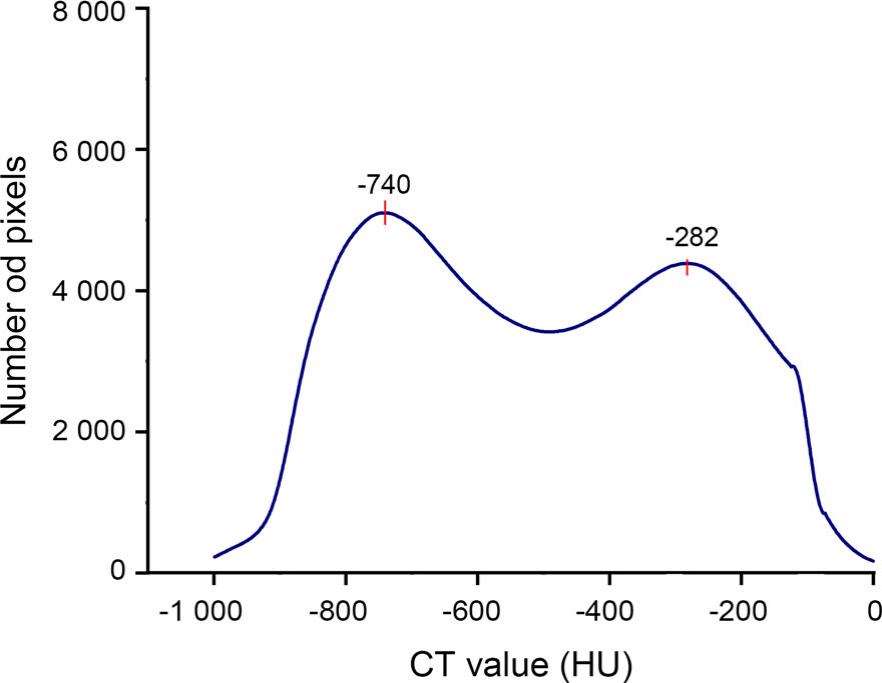
The mean distribution curves in coronary artery disease. The curve presents two broad peaks after a rapid rise at −740 and −282 HU. The corresponding number of pixels of the peaks are 5 105 and 4 386, respectively.

Lung CT value distribution in drowning presented a bell-shaped curve (Figure 4), peaking between −800 and −400 HU. In the control group, there were two different patterns (Figure 5). One was similar to the drowning group’s bell-shaped curve in three of 12 cases, with peaks appearing at −713, −550 and −250 HU, respectively. In the other nine control cases, the lung CT value distribution curves were relatively flat. But these curves still showed two lower peaks between −900 and −700 HU and between −300 and −150 HU, except in Cases 7 and 12. According to the mean distribution curve in drowning (Figure 6), the curve was unimodal, peaking at −659 HU. The control group’s mean curve was bimodal (Figure 7), with peaks at −740 and −282 HU.

## Discussion

According to previous studies, GGO is found frequently on postmortem chest CT image in drowning. Usui et al. [14] evaluated the features and further classified GGO into six types. However, GGO also has been described in other causes of death, especially cardiac death [15,16]. In this study, GGO was found on postmortem chest CT image in drowning and most cases of the control. The authors attempted to find more differences between drowning and non-drowning cases via PMCT data analysis.

Drowning can be divided into two main types: wet drowning and dry drowning. The former predominates, accounting for approximately 90% of drowning [17]. The major difference between wet and dry drowning is fluid aspiration in the lung. In autopsy, emphysema aquosum is a common and classical finding, which describes hyperinflation of the lungs caused by inhaling a mixture of air and fluid. Sometimes, indentations from the ribs can be found on the surface of the lungs. When the lungs are cut, red transparent fluid with white foam flow from the cut surface. To evaluate the degree of inflation of the lungs, previous studies used two measurements. One is the anterior interpulmonary distance, another one is the anterior rib level of the right hemi-diaphragm. However, these changes caused by inflation of the lungs were not obvious, even though the difference was only approximately one rib level. Because of individual variation and the small absolute difference, its use is questionable in forensic practice [12]. In order to evaluate more accurately, our team estimated the volume of the whole lung. Compared with Sogawa et al.’s studies [18], the mean value of lung volume in drowning was similar. According to the result at present study, lung volumes were not significantly different between the drowning and the control group, probably due to the limitations of sample size and the influence of individual factors (such as the primary lung volume). Considering the varying of lung volume caused by individual factors, our team attempted to assess the change of the lungs with another indicator, lung volume ratio.

Lung volume ratio, which was posited as an indirect indicator of inflation of the lungs, was significantly larger in drowning than in control cases. The lungs become blunter and more full caused by hyperinflation due to inhaling an amount of foamy fluid into the lungs, especially the unlimited or flexible parts (such as apex and base). Because of the limitation of thoracic wall, the influence on the maximum volume was smaller than lung volume. So, compared with other causes of death, lung volume ratio (lung volume/maximum volume) in drowning is larger due to the expansion. There was an overlap of lung volume in drowning and controls. The explanation may be individual variation or the differences in the quantity of inhaled water. In this situation, we suggest that lung volume ratio, but not lung volume, be used as an indicator to evaluate the degree of inflation of the lungs.

The lung density is mainly determined by the lung parenchyma, blood in small vessels and air in the lung. The proportion of the three varies with physiological activities, which leads to changes in CT values [19]. Sogawa at al. [18] found pulmonary air/gas content in drowning was higher than that in sudden cardiac death. The victim breathes heavily during drowning, but the air in the lungs is difficult to exhale. Bronchial constriction caused by inhaled water and foamy fluid increase the resistance to exhalation. However, there was no significantly difference between the drowning and the control group in the mean CT value of the whole lung. The explanation could be the inhaled fluid weakens the influence of the air on the mean CT value of the whole lung.

There was a difference between drowning and control groups in the mean CT attenuation value distribution curves. The CAD curve displayed a bimodal distribution after a rapid rise, but only one peak was found in drowning. In a previous report, the CT value range, from −1 000 to −700 HU, was used to assess the normal aerated lung [20]. Compared with the normal aerated lung, the lung CT value increased both in drowning and control group. Replacement of air by fluid or cells and an increase in the blood volume both lead to CT value increase [19]. We consider that the first peak is associated with pulmonary oedema, in which the air of alveoli is replaced by fluid or cells. Severe pulmonary congestion, which is a common pathological change in CAD, causes the increase in the blood volume. It could be the reason behind the second peak in the CAD cases. The density of the blood should be higher than fluid and cells, which lead to the greater increase in lung attenuation. So another peak appeared later than the first one. The degree of pulmonary congestion has an effect on the shape of the second peak to some extent, even leading to only one peak in the curve (Cases 11 and 18). From the above, we suggest that the lung CT value distribution could be a potential reference index to distinguish between drowning and CAD. Two peaks tend to appear in the curve of CAD, and one in drowning.

Lung 3D reconstruction would be an interesting approach to apply in forensic diagnosis. The data extraction is simple and quick (about 30 min). The first limitation of our study is the small sample size. In our institution, severe postmortem decomposition occurred in a large proportion of cadaver when retrieved from the water. This limitation has an effect on statistically significant results to some extent. Further study is guaranteed based on a larger sample size. Secondly, the victims all are died of typical drowning in fresh water in this study. The study did not take the other pathomorphology of drowning (atypical, combined) into consideration. Maybe, there is difference in cases of fresh water drowning and drowning in salt water.

## Conclusion

PMCT of the chest is helpful for forensic medical diagnosis of drowning. Lung volume ratio and lung CT value distribution may be potential indicators to distinguish between drowning and CAD.

## Compliance with ethical standards

This article does not contain any studies with human participants or animals performed by any of the authors.
